# Annotations

**Published:** 1899-09-23

**Authors:** 


### ANNOTATIONS.

### The British Association.

THE meeting of the British Association for the Advancement of Science is always an occasion which affords opportunity to the members of the medical profession to illustrate at once the prominence of their calling in respect of the cultivation of science in general, and the vast importance to the public of those departments of inquiry which fall more especially into the hands of the professors of the healing art. The meeting at Dover this year has manifestly been conducted under some difficulties, for which due allowance must be made. The town itself is small for the purpose, and could hardly be expected, without some strain on its resources, to provide accommodation for the visitors, usually about two thousand in number, who are accustomed to attend. When the association goes to Manchester or to Liverpool its constituent units are absorbed, almost unnoticed, into the mass of the population. At Dover, even with full recollection of the receptive capacities of neighbouring localities, such as Deal, Walmer, and St. Margaret's, this could hardly be the case; and it need occasion no surprise that the comparatively few lodging-houses in the town, the owners of which would in all probability regard every branch of science with equal indifference, should only be obtainable at higher rates than the members of the association were accustomed to pay. We will not accuse the local committee and the local secretaries of any undue tenderness towards the expectations of the landlady class; but it seems certain that they did not go quite the best way to work, and that their arrangements, made through the intermediation of house agents, were not entirely successful in their working. The reports of the papers read which have appeared in the daily press are scarcely sufficient in themselves to afford materials for a very accurate determination of their relative or respective merits, and it is manifest that the most noteworthy of these papers would be likely to require a more critical consideration than would be possible, even after having heard them when delivered. But a very prominent place, if not the first, at least among those dealing with any biological question, should probably be accorded to Mr. Langley's address in the section on physiology, in which he expounded views concerning the functions and relations of different portions of the nervous system which depart materially from some of those hitherto entertained, and which, as he himself admitted, cannot yet be considered as entirely established. His main contention was that large portions of the organism are less dependent upon nervous action or stimulation than has hitherto been believed; that they live their own lives uninfluenced, except indirectly, by the storms and stresses of the central nervous system. No nervous impulse can pass to them to make them contract or to make them secrete, or to quicken or slacken their inherent activity. The nervous system can only influence them through the medium of some other tissue by changing the quantity or quality of the surrounding fluid. As one illustration, Mr. Langley mentioned the case of the sub-maxillary gland, which not only at fitting seasons pours saliva into the mouth, but is also during life ceaselessly taking in oxygen and giving out carbonic acid, and does this without pouring forth any attendant secretion. So far as we know, he tells us, no nervous impulse can hasten or retard this customary life of the gland by a direct action upon it without producing other changes. He describes the essential effect of a nerve impulse as modifying the amount of energy set free as work, either by increasing or by diminishing it, and, although other changes may go on side by side with this setting free of energy, there is no unimpeachable instance in which they take place by themselves as the results of nervous excitation. It is manifest that these views, if confirmed, are likely to exert no small influence upon many aspects of medical doctrine.

### The Mosquito Hunt at Sierra Leone.

THE two most interesting points brought out by the researches now being made by the malaria expedition to Sierra Leone are, first, the amount of mischief which may be done by a comparatively small number of infected mosquitoes; and, second, the important part played by malaria-infected man in spreading the disease. Putting aside the question of what is the original and natural home of the plasmodium, the question, that is, as to the sort of animal other than man in whose blood it naturally grows, what has now been made quite clear is that a malaria-infected man is a distinct danger to his healthy fellows so long as the particular sort of mosquito (*anopheles*) which is capable of carrying the parasite is present. Malaria, in fact, is a very definitely infectious fever, but the infection must be carried the right way, namely, by the particular mosquito in whose body it can undergo certain phases of its development. Not only then do patients catch malaria from each other, viâ the mosquito, but it even happens that they become constantly reinfected afresh, maybe from themselves, by aid of this infection carrying insect. It is easy then to see how it is that in certain houses or groups of houses which have once become infected the disease becomes endemic. But, although endemic, it is still an infection, the endless cycle always being from man to mosquito, from mosquito to man. The number of insects actually engaged in this nefarious business may not be great. It appears that already the hunt for *anopheles* in the barracks at Wilberforce set on foot by Dr. Donald Ross and his party has so diminished their numbers that they are beginning to be hard to find, and it seems probable that persistent efforts to destroy

**ANNOTATIONS**—*continued.*

these injurious blood-suckers and the careful use of mosquito nets at night may prove of immense effectiveness in lessening the prevalence of malaria in certain notoriously malaria haunted localities.

### Funeral Scarves and Secret Commission.

At last a medical man has stepped forward to declare that he has been offered a "secret commission." "Some years ago," he says, "I received a cheque from an undertaker 'in lieu of scarves for the funerals of A. B. C., &c.,'" the cheque amounting to six or seven guineas. Now, if this was a secret commission, what about the scarves themselves? The whole thing is absurd, almost as absurd as the custom of wearing these scarves. It used to be customary, and still is we fancy in some places, for the friends and relatives attending a funeral to bedeck themselves in black silk scarves; and when the doctor was asked to a funeral he, like every other invited guest, was rigged up for the occasion with a scarf, and a brand-new pair of black kid gloves was thrust into his hands. All these things were charged in the undertaker's bill, and there was no secrecy about it. So rigorous, indeed, was the custom in some parts of the country of wearing this wretched thing in church the Sunday afterwards that, even if the doctor was prevented attending the funeral, the scarf, that great emblem of a respectable burying, would be sure to arrive at his house in due course. Was this a commission? Not a bit of it. It was a frightful nuisance. Although in our opinion the doctor did quite right to refuse the cheque, we are quite sure the undertaker never looked upon it as a "secret commission." Good, honest man, he knew that his bill had contained the item, "to so many scarves, so much"; that the executors had, in fact, paid him for a scarf to be sent to the doctor; and that in sending on a cheque worth about half the value of the silk he was doing real good business.

### The Polyclinic.

At last the Medical Graduates' College is able to show work done. That its managers have been somewhat slow in getting their lectures and demonstrations into full working order has been partly due, perhaps, to their ambitions, and to their desire to place the college on a firm foundation before promising too much. But with the appearance of the second number of the *Polyclinic*, the monthly journal of the college, we see proof that a really useful and practical attempt to supply the practitioner with post-graduate clinical instruction is now in full working order. The college does many things. It has courses of lectures and practical classes, especially the latter, on all sorts of subjects. But the most interesting feature of its work is the establishment of daily consultations, clinical demonstrations on cases which are so grouped as to illustrate special forms and phases of disease. This is a side of the work which undoubtedly will grow, for it offers to all practitioners a means of obtaining an expert opinion on curious or difficult cases they may meet with in patients who are not able to afford consultation fees. All they have to do is to communicate with the medical superintendent of the college, who will arrange as to the day and hour when the case may best be considered. Of course, everyone to his taste; some like reading, others hearing, others seeing, and the brains of some are more easily accessible by one route than another, but we cannot but feel that not only should the Medical Graduates' College score a considerable success among medical visitors from the provinces, the colonies, and from foreign countries, but that it should become part of the professional life of all medical men who work in London. Nothing could tend more to keep a man in touch with medical progress than the spending of one afternoon a week at the Polyclinic; sometimes attending classes, sometimes consultations, but always devoting that afternoon to the duty of increasing his knowledge and to the pleasure of meeting his professional brethren. The college ought not only to be a house of call for medical strangers, but it ought also to become a central point of union for all that is best among the general practitioners in the London district.

### Training for Life Insurance.

The last of the accusations cast in the teeth of the medical profession comes from New York, where, we are told, certain physicians are said to thrive on the practice of assisting rejected candidates for life insurance to get into such physical condition as will enable them to pass the medical examination, and so victimise the companies. There is a fine amateur flavour about this little tale. Unfortunately for suffering mankind, however, the human frame is not so easily "faked," nor will any pharmaceutical "putty and paint" make it "equal to new," as the phrase goes among the coach builders. And so we venture to disbelieve the whole story. We are not concerned to deny that a slight degree of diabetes or albuminuria might be masked by a short course of appropriate diet, or that by knocking off a man's whiskey and tobacco, or even his tea, a fluttering heart may be made to run steadily. These things come under the head of treatment, are for the patient's good, are perfectly legitimate, and have no bearing on the question of insurance unless accompanied with a nice little assortment of lies, which an insurance company (in England, at any rate) is certain to find out. Various questions very pertinent to this matter appear on most English proposal forms, such as "When did you last consult a doctor, and for what complaint?" and the answers to these questions become part of the proposal, any untruth in which invalidates the policy. We do not deny that a man who suspected he was failing, especially if he happened himself to be a physician, might in some rather rare cases "doctor himself up" so as to pass a careless examiner; but we entirely disbelieve that there are physicians who make a business of doing this for their clients.

### Liverpool Fever Hospital.

In addition to five municipal hospitals for infectious disease in various parts of the city the Liverpool Corporation possesses an estate of 113 acres at Fazakerley, for which they gave £40,000, on which are to be erected a convalescent hospital and two small-pox pavilions, with 20 beds in each. A curious combination, which, however, will have this advantage, that it will necessitate the vaccinating of the convalescents. After a recent inspection of the city hospitals, Dr. Hope, medical officer of health, referring to the benefit that they had been to the community, stated that whereas 15 years ago there were 500 typhus fever cases a year they had now fallen to 26.

